# Impact of the COVID-19 Pandemic on the Work Activity of Spanish Physical Therapists and Their Response to Vaccination

**DOI:** 10.3389/fpubh.2022.877232

**Published:** 2022-05-12

**Authors:** Sergio Montero-Navarro, Jesús Sánchez-Más, Cristina Salar-Andreu, Francisco Javier Molina-Payá, Cristina Orts-Ruiz, José Martin Botella-Rico, José Tuells, Noelia Rodríguez-Blanco

**Affiliations:** ^1^Nursing and Physical Therapy Department, Health Sciences Faculty, CEU-Cardenal Herrera University, CEU Universities, Elche, Spain; ^2^Biomedical Sciences Department, Health Sciences Faculty, CEU-Cardenal Herrera University, CEU Universities, Elche, Spain; ^3^Department of Community Nursing, Preventive Medicine and Public Health and History of Science, University of Alicante, Alicante, Spain; ^4^Department of Obstetrics and Gynaecology, Marina Baixa University Hospital, Villajoyosa, Spain

**Keywords:** vaccines, physical therapists, COVID-19, SARS-CoV-2, acceptance, impact

## Abstract

Due to the COVID-19 pandemic, physical therapists have had to adopt a set of specific protection measures, which have had an impact on their clinical activity and economy. The objective was to evaluate the impact of the COVID-19 pandemic on the work of Spanish physical therapists, as well as their attitudes and predisposition to vaccination. An online questionnaire was divided into five sections: (1) demographic and professional data; (2) labor impact; (3) precautions and infection-control measures; (4) economic impact; and (5) vaccine acceptance and adverse effects. Of the 666 participants, 62.1% showed a reduction in their working hours motivated by: fear of infection (*p* = 0.007), financial issues (*p* = 0.002) and being in quarantine or isolation (*p* < 0.001). Of these, 36.4% were forced to close the clinic, 62.7% requested help from the government, but only 12.04% mentioned that it was adequate. The main prevention measures adopted were the use of gels and masks and, in the private sector, disinfection with ozone or ultraviolet light (*p* < 0.05). The acceptance of the vaccine was high, 87.5%, being lower among the group over 40 years of age, self-employed, widowed or separated. More adverse effects were mentioned after receiving the AstraZeneca-Oxford vaccine, compared to Pfizer-BioNTech or Moderna. In conclusion, this study assessed for the first time that the COVID-19 pandemic in Spain had a negative impact on work and finances of physical therapists. The vaccine was widely accepted, in part by the economic impact that an infection in the work setting could signify.

## Introduction

On March 11th, 2020, the World Health Organization (WHO) declared a world pandemic due to the fast, worldwide propagation of the virus named SARS-CoV-2, which produced the disease named COVID-19 (1). From that moment on, Spain was one of the most-affected countries, with a total number of 87,462 confirmed deaths and 5,019,255 confirmed cases (2, 3). Spain announced a state of alarm on March 14th, 2020, through the Royal Decree 463/2020 (4), which included restrictions such as limitations on movement of people, cancelation of public events, isolation and quarantine, among others.

The expansion of the virus created a saturation in the health system, placing the collective of health professionals, among which we find physical therapists, in a very complicated situation (5–7). The physical therapist clinics could only remain open to tend to emergencies, without being able to close, as they were considered essential health professionals. Also, given the close physical contact between the patient and the therapist (8, 9), the physical therapists had to adopt a set of specific protection measures to perform their professional care activities, such as: the use of personal protection equipment (PPE), telephone-based triage, questionnaire for the detection of COVID-19 patients, reduction of patients/day, disinfection of hands, ventilation between patients, limits in companions, use of aerosol filters, among others. This situation had an enormous economic impact on the private clinics, who had to absorb the cost associated with these new measures. Also, the fear of the population of becoming infected notably reduced the activity of these physical therapy clinics (10). On May 4th, 2020, a de-escalation plan approved by the government was initiated, which progressively reduced the restrictions and lasted until September 2020 (11). However, it was not until May 9th, 2021, that the state of alarm ended (12).

In the meantime, the restrictions were modified as a function of the health situation associated to the COVID-19 pandemic, and therefore, the clinics also had to adapt to guarantee the safety of the patients and workers. Faced with this scenario, many of the Spanish physical therapists who worked in private clinics decided to close their clinics and ask for State-funded aid (13). However, the concession of these aid packages was not ensured, as the Spanish Government established that private health clinics were an essential activity that must remain open, although they were only authorized to provide emergency care, as indicated in the Royal Decree 09/2020 from March 28th (14), and the Royal Decree 10/2020 from March 29th (15).

On the other hand, the only strategy that was able to stop the spread of the virus was to achieve herd immunity in the entire country, either through vaccination, natural infections, or a mix of both (16). In Spain, the vaccination campaign begun on December 27th, 2020, and according to the vaccination strategy, the first to receive it would be individuals who were institutionalized and its workers, front-line health professionals, social health workers, and dependent individuals (3). As for the physical therapists, the Spanish government started the vaccination of those who worked in the public health system, who were provided the Pfizer-BioNTech (PB) vaccine.

After the vaccination of the public health physical therapists, the private mutual insurance companies managed the administration of the vaccines to the workers who worked in private healthcare, who received the AstraZenexa-Oxford (AZ) vaccine. During this period, physical therapists were also given an appointment for vaccination according to their age groups and were able to receive any of the available vaccines. This management resulted in differences among professionals, in both the date of vaccination and the type of vaccine received. Afterwards, on March 16th, 2021, the Spanish government decided to temporarily suspend the vaccination with AZ due to the appearance of cerebral thrombosis and other secondary effects (17), with a great number of private healthcare physical therapists without a first dose or without being able to complete the vaccination against COVID-19. The vaccination campaign resumed a few weeks after, and the physical therapists with the first AZ dose were allowed to choose between AZ or PB to complete the vaccination schedule, under informed consent (18). This led to uncertainty in the physical therapist collective, which led to increased reticence and the decrease in the acceptance of the second dose of the vaccine among Spanish physical therapists.

In this regard, it is necessary to assess in Spain the impact of the pandemic due to COVID-19 on physical therapists during the state of alarm decreed by the Spanish government, to discover the decisions and measures adopted by these professionals associated with the work setting and their decision to become vaccinated.

## Materials and Methods

### Design, Population, and Sample

A cross-sectional survey was conducted using an electronic questionnaire that was electronically sent to Spanish physical therapists by the General Council of Physical Therapist Associations of Spain, starting on June 8th, 2021, and ending on June 28th, 2021. The total number of registered Spanish physiotherapists in 2021 was 45.061, which was used as the reference population. The calculated sample size was equal to 381, with a level of confidence of 95%, and a margin of error of 5% (19, 20).

### Data-Collection Tool

A questionnaire designed *ad hoc*, based on a previously published survey (21), was utilized as the data-collection instrument, and a pilot study was conducted with a group of 25 physical therapists, who were not considered for this analysis. This pilot study allowed us to collect the impressions of those polled, and the questionnaires were afterwards evaluated by a group of experts to assess the understanding of the questions by the respondents and to determine how long it took them to complete it, and to check the internal consistency of all questionnaire's components. The final questionnaire was composed of 23 closed-ended or ten-point Likert scale questions.

These questions were divided into five sections: (1) six questions designed to collect demographic and professional data (age, sex, marital status, employment, years of work experience, professional role); (2) two questions about the impact in the professional sector and the triggers; (3) two questions about practice modification, the precautions and infection-control measures against COVID-19 infection; (4) five questions assessed the economic impact in the health care activity, and the soliciting and concession of State-funded aid; and (5) eight questions about the impact of COVID-19, acceptance of the vaccine, adverse effects of the vaccine, and reasons for accepting the vaccine. The voluntary consent, objectives of the study, the code of acceptance from the ethics committee, and the estimated length of time needed to complete the questionnaire were included on the questionnaire heading. A total of 692 questionnaires were obtained, with a final sample of 666 due to the exclusion of 26 incomplete questionnaires.

### Methods of Analysis

The mean ± standard deviation was utilized for the quantitative variables, and frequency tables for the qualitative data. The Chi-square test was utilized to investigate the relationships between the categorical variables. The factors associated to the willingness to receive the vaccine were identified through the use a logistic regression analysis. A multivariate logistic regression was performed with the 581 participants who claimed to have received the vaccine, in order to identify the adverse effects associated with the type of vaccine received, with the odds ratio (OR) probability and a confidence interval (CI) of 95% calculated. The likelihood was calculated with the Wald Chi-square test, and the goodness-of-fit was tested with Pearson's test. The statistical analysis was performed with the IBM SPSS Statistics software program for Windows, version 24.0.

### Ethical Considerations

The study was conducted in accordance with the principles from the Declaration of Helsinki on human clinical trials (Ref No. 579/06/2020) and was approved by the Ethics Committee from the CEU Cardenal-Herrera University (CEI21/060).

## Results

### Demographic and Professional Data

A total of 666 physical therapists completed the questionnaire, of which 72.1% (*n* = 480) were women. Their mean age was 32.8 years old, and 69.4% (*n* = 462) lived with a partner. Their mean work experience was 9.8 years, with a distribution of mainly private sector workers, 90.3% (*n* = 602), including freelancers and self-employed individuals. With respect to the professional role, 15.2% (*n* = 101) were employers, and the rest employees or freelancers (Table 1).

**Table 1:** Demographic and professional data of the physical therapists (*n* = 666).

**Variables**	**Findings**
Sex	
Female	480 (72.1)
Male	186 (27.9)
Age (years)	32.8 ± 7.0
Age group	
20–29 years	254 (38.1)
30–39 years	281 (42.2)
>40 years	131 (19.7)
Marital status	
Single	185 (27.8)
Married	193 (29.0)
Living with a partner	269 (40.4)
Divorced/Widowed	19 (2.8)
Work experience (years)	9.8 ± 6.9
Group work experience	
<5 years	190 (28.5)
5–10 years	192 (28.8)
11–15 years	138 (20.7)
>16 years	146 (21.9)
Type of employment	
State / interim worker	44 (6.6)
Freelancer	324 (48.6)
Self-employed	278 (41.7)
Sick leave /unemployed	20 (3.0)
Professional role	
Employer	101 (15.2)
Employee/freelancer	556 (83.5)
No answer	9 (1.4)

*Data shown as number of responses (percentage)*.

### Impact on the Physical Therapist Work During the Pandemic

**Figure 1:**
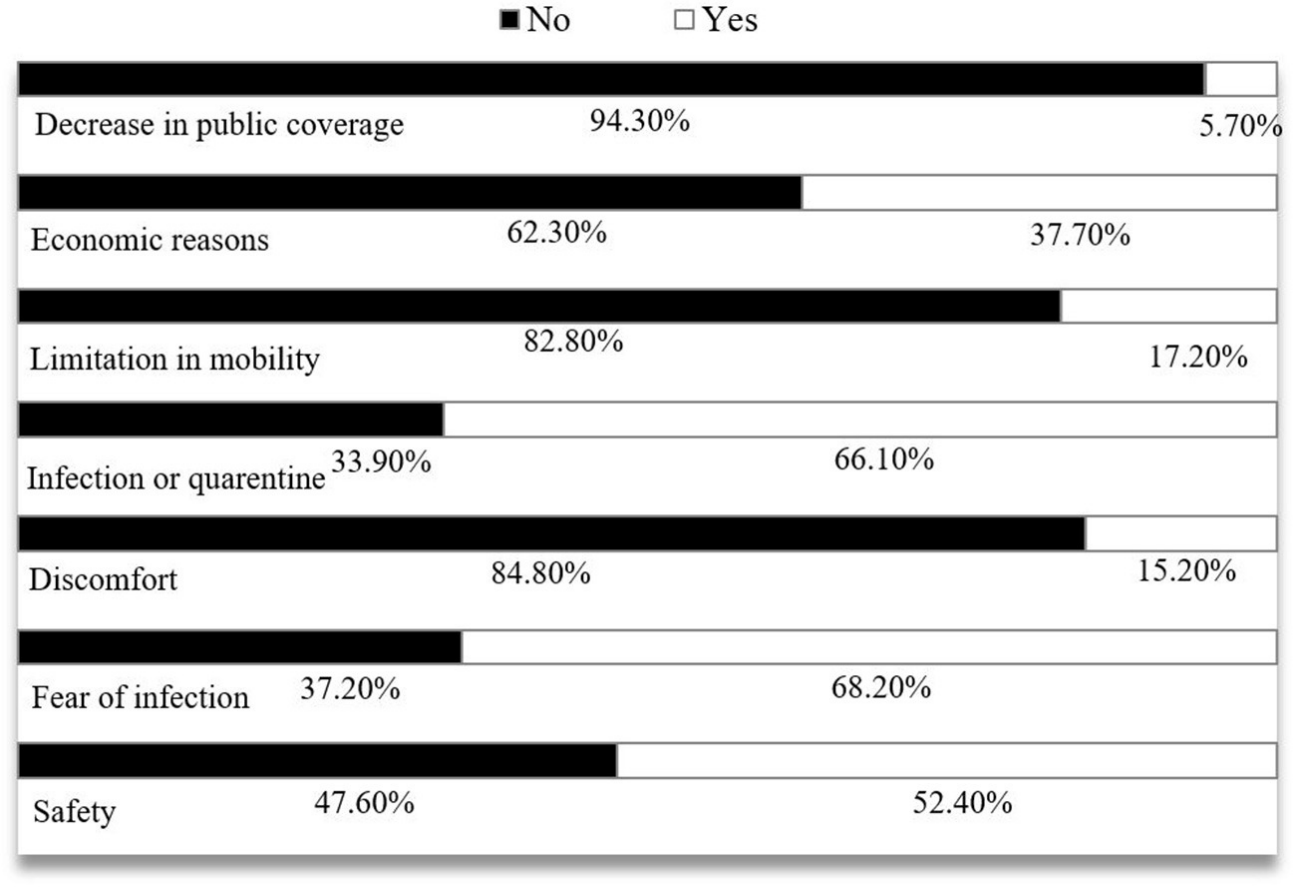
Reasons provided by the physical therapists that resulted in the reduction of scheduled appointments during the pandemic.

From the total participating physical therapists, 62.1% (*n* = 401) mentioned a reduction in their scheduled appointments, as compared to 31.6% (*n* = 204) who kept the same number of patients, despite the restrictions and conditions marked by the pandemic. With regards to those who indicated a reduction in their work schedule, they were asked for the reasons behind this change (Figure 1).

The main reasons were; fear or worry of the patients of becoming infected at the clinic (68.2%), being in quarantine due to a close contact with a COVID-19 case, or due to the infection of the patient (66.1%). Other reasons provided were the decision to reduce the appointment schedule to guarantee the safety of the patients and workers (52.5%), the limits placed on mobility (17.2%), or economic reasons (37.7%).

Among the physical therapists who affirmed to have suffered a reduction in appointments (*n* = 401), 92.0% (*n* = 369) worked in the private sector, and 8.0% (*n* = 32) in the public one. The statistical analysis showed that the reduction in the work schedule of the physical therapists who worked in the private sector, as compared to those who worked in the public sector, was greatly influenced by the variables “fear of infection of the patient” (*p* = 0.007), “the discomfort of the patient with the new safety measures implemented” (*p* = 0.047), “economic reasons” (*p* = 0.002), and due to “quarantine or infection of the patients” (*p* < 0.001). The rest of the statements did not show significant differences between the public and private sectors.

### Modification of Practices Associated to the Precautions and Infection-Control Measures Against COVID-19 Infection

The PPE, the hygiene measures, and the safety measures incorporated by the physical therapists who worked in the private and public sectors are shown in Table 2. For the physical therapists who remained active, the main PPEs and hygiene measures incorporated to the clinical practice were the use of alcohol-water gels (95.7%) and the FFP2 or FFP3 masks (92.7%). Other measures implemented were the use of surgical masks (65.8%), gloves (64.7%), and protection goggles or screens (57.0%), as well as leaving a period of time between patients to ventilate and disinfect the room (74.0%). The measures of use of ozone (9.4%) or ultraviolet light (3.4%) were less-utilized by the physical therapists, as well as the periodic testing for COVID-19 through PCR or antigen tests, by neither the companies (12.1%) nor the physical therapist (10.4%).

**Table 2:** Personal protection equipment, hygiene measures, and safety measures incorporated at work during the pandemic by the physical therapists.

	Total^**^(*n* = 646)	**Private sector (*n* = 602)**	**Public sector (*n* = 44)**	**Chi^**2**^ value**	***p***
**Personal protection equipment incorporated at work**					
FFP2 or FFP3 masks	599 (92.7)	557 (92.5)	42 (95.5)	0.52	0.470
Surgical masks	425 (65.8)	396 (65.8)	29 (65.9)	<0.01	0.986
Gloves	418 (64.7)	393 (65.3)	25 (56.8)	1.29	0.257
Goggles or protection screens	368 (57.0)	343 (57.0)	25 (56.8)	<0.01	0.984
Disposable gown	276 (42.7)	259 (43.0)	17 (38.6)	0.32	0.570
Disposable shoe covers	113 (17.5)	105 (17.4)	8 (18.2)	0.02	0.901
Disposable caps	103 (15.9)	92 (15.3)	11 (25.0)	2.89	0.089
**Hygiene or safety measures incorporated at work**					
Water-alcohol gels	618 (95.7)	577 (95.8)	41 (93.2)	0.70	0.402
Time between patients	478 (74.0)	446 (74.1)	32 (72.7)	0.04	0.843
Not seeing two patients at the same time	264 (40.9)	247 (41.0)	17 (38.6)	0.10	0.755
Ventilation and disinfection of the rooms	253 (39.2)	237 (39.4)	16 (36.4)	0.16	0.693
Air filtration equipment	151 (23.4)	143 (23.8)	8 (18.2)	0.71	0.399
PCR test of oneself	67 (10.4)	67 (11.1)	0 (0.0)	5.46	0.019 ^*^
Company provides PCR test	78 (12.1)	75 (12.5)	3 (6.8)	1.23	0.268
Disinfection with ozone	61 (9.4)	61 (10.1)	0 (0.0)	4.92	0.026 ^*^
Ultraviolet light	21 (3.3)	21 (3.5)	0 (0.0)	4.34	0.046 ^*^

*Data shown as n (%)*.

* *p <0.05 Chi square calculated as contingency table*.

** *Population calculated after eliminating those who were sick leave or unemployed (n = 646)*.

The statistical test only showed significant differences between the private and public sectors with respect to the use of ozone or ultraviolet light disinfection, as well as one's own decision to take a PCR test.

### Economic Impact of the Health Care Activity of the Private Sector Physical Therapists

The participants were asked about the economic impact of the pandemic experienced by the physical therapists who worked in the private sector (*n* = 602) during the state of alarm period, from March 14th, 2020 to May 9th, 2021. Of the participants, 36.4% (*n* = 219) was forced to close the clinic during the pandemic periods in which work was allowed. Also, of the 62.7% (*n* = 377) who solicited the economic aid from the Spanish government, most were granted, except for 8.0% which were rejected. When analyzing the opinion of the physical therapists who received the aid, 12.04% (*n* = 72) affirmed that the aid completely covered the economic impact during the state of alarm period, while 56.3% (*n* = 339) indicated that it was not sufficient.

Finally, when asked about the economic impact associated to the pandemic period according to a Likert scale, which range from 0 (no economic impact) to 10 (maximum economic impact), the physical therapists who worked in the private sector referred to an economic impact of 5.9 ± 2.5 with respect to the previous years.

### Attitudes Toward Vaccination and the Current State of Health

As for the impact of COVID-19 infection between the participating physical therapists, 15.5% (*n* = 103) declared that they had recovered from the disease, and 32.9% (*n* = 219) had to be quarantined at home due to infection or being a close contact with a person infected with COVID-19 (Table 3).

**Table 3:** Impact of COVID-19, vaccine acceptance and adverse effects of vaccination.

**Variables**	**Findings**
Have you been infected with COVID-19?	
Yes	103 (15.5)
Were you quarantined?	
Yes	219 (32.9)
Have you received the COVID-19 vaccine?	
No	85(12.8)
Rejected	35 (5.3)
It was not offered to me	50 (7.5)
Yes	581 (87.2)
What vaccine was used as the first dose? (*n* = 581)	
AZ	406 (61.0)
PB	141 (21.2)
Mo	34 (5.1)
Did you receive a second dose? Which one (*n* = 581)	
No	221 (38.0)
Waiting	136 (61.5)
It was not offered to me	50 (22.6)
Rejected	35 (15.8)
Yes	445 (62.0)
AZ	259 (58.2)
PB	161 (36.2)
Mo	25 (5.6)
Any adverse effects after vaccination? (*n* = 581)	
Yes	528 (90.9)
Pain in the arm	471 (81.1)
Tiredness	365 (62.8)
Fever	290 (49.9)
Headache	274 (47.2)
Generalized muscle pain	268 (46.1)
Shivering	245 (42.2)
Inflammation of the ganglia	47 (8.1)
Vomiting	31 (5.3)
Would you accept a second dose? (*n* = 115)^*^	
Yes, even if it's a different brand	28 (24.3)
Yes, but only if it's the same brand	80 (69.6)
No, under no circumstances	4 (3.5)
I don't know	3 (2.6)

*Data shown as n (%)*.

* *percentage calculated with respect to the total number of participants who received the first AZ dose and were waiting to receive the second one. AZ, AstraZeneca-Oxford; PB, Pfizer-BioNTech; Mo, Moderna*.

The acceptance of the vaccine was greatly favorable, as 87.2% (*n* = 581) indicated having received the AZ, PB, or Moderna (Mo) vaccines. Most were vaccinated with AZ (61.1%) in the first round, with PB being the second most-common vaccine administered (21.2%). With respect to the second dose, 20 more physical therapists received PB in the second round instead of AZ. There were 136 participants (20.4%) who were still waiting for the second dose. Of these, 10 participants received PB in the first dose, 11 received Mo, and 115 received AZ. Of the total number of participants vaccinated with the AZ vaccine as a first dose, most would accept the same vaccine only if they received the same one (69.6%), and only 28 participants (24.3%) would receive a different one. A rejection of the second dose was observed in 3.5% of those who were vaccinated with the first dose of AZ.

The most frequent adverse effect among those vaccinated was pain in the place where the vaccine was administered (81.1%), followed by tiredness (62.8%). The percent was calculated considering that only 581 indicated having been vaccinated. The adverse effects with a frequency close to 50% were fever (47.2%), headache (47.2%), generalized muscle soreness (46.1%), and shivering (42.4%). The inflammation of ganglia or vomiting were observed with a frequency lower than 10%. Only 53 participants (9.1%) indicated not suffering from any of the symptoms mentioned previously.

The multivariate logistic regression performed with the 581 participants who had received the vaccine showed that the administration of the AZ vaccine was associated with a greater appearance of headaches (*p* = 0.002) and shivering (*p* < 0.001) with respect to the administration of the PB vaccine (Table 4).

**Table 4:** Multivariate logistic regression analyses showing adverse effects associated to the vaccine brand received by the physical therapists who participated in the study.

**Adverse effects**	**AZ (*n* = 406)**	**PB (*n* = 141)**	**OR (95% CI)**	***p***	**Mo (*n* = 34)**	**OR (95% CI)**	***p***
Pain at the place where the vaccine was administered							
Yes	330 (81.3)	112 (79.4)	1.61 (0.77–3.36)	0.203	29 (85.3)	0.93 (0.30–2.90)	0.894
Fever							
Yes	231 (56.9)	44 (31.2)	0.67 (0.40–1.13)	0.132	15 (44.1)	1.43 (0.60–3.40)	0.421
Headache							
Yes	219 (53.9)	42 (29.8)	0.49 (0.31–0.77)	0.002^*^	13 (38.2)	0.69 (0.32–1.50)	0.351
Vomiting							
Yes	28 (6.9)	2 (1.4)	0.48 (0.11–2.13)	0.332	1 (2.9)	1.22 (0.14–10.5)	0.858
Tiredness							
Yes	264 (65.0)	82 (58.2)	1.48 (0.89–2.44)	0.129	18 (52.9)	0.92 (0.42–2.05)	0.844
Shivering							
Yes	208 (51.2)	29 (20.6)	0.34 (0.20–0.58)	<0.001^*^	8 (23.5)	0.32 (0.12–0.82)	0.017^*^
Generalized muscle pain							
Yes	212 (52.2)	50 (35.5)	0.90 (0.54–1.49)	0.678	6 (17.6)	0.23 (0.08–0.62)	0.004^*^
Inflammation of the ganglia							
Yes	36 (8.9)	6 (4.3)	0.56 (0.22–1.44)	0.229	5 (14.7)	0.12 (0.01–1.22)	0.353

*Data shown as n (percent within the vaccine brand). OR = calculated for each answer as compared to all the others; p = calculated for the Chi-square test group. The negative response to the presence of the adverse effect was taken as the reference value for the multivariate logistic regression analysis. AZ, AstraZeneca-Oxford; PB, Pfizer-BioNTech. Mo, Moderna*.

* *p <0.05 Chi-square calculated as contingency table*.

Likewise, the administration of the AZ vaccine was associated with a greater appearance of shivering (*p* = 0.017) and generalized muscle soreness (*p* = 0.004) with respect to the administration of Mo. For the rest, the adverse effects did not show significant differences according to the vaccine received.

The analysis of the influence of the sociodemographic or work characteristics on the acceptance or rejection of the COVID-19 vaccine by the participating physical therapists is shown in Table 5. Thus, the physical therapists who rejected the COVID-19 vaccine to a higher extent were older than 40 years old, divorced/widowed, and freelancers.

**Table 5:** Profile of the physical therapist who accepts or rejects the COVID-19 vaccine.

**Variables**	**Total (*n* = 616)**	**Acceptance group (*n* = 581)**	**Rejection group (*n* = 35)**	**Chi^**2**^ value**	***p***
Sex				0.30	0.586
Female	447 (72.6)	423 (94.6)	24 (5.4)		
Male	169 (27.4)	158 (93.5)	11 (6.5)		
Age group				10.14	0.006^*^
20–29 years	230 (37.3)	223 (97.0)	7 (3.0)		
30–39 years	261 (42.4)	247 (94.6)	14 (5.4)		
>40 years	125 (20.3)	111 (88.8)	14 (11.2)		
Marital status					
Single	166 (29.6)	161 (97.0)	5 (3.0)	27.76	<0.001^*^
Married	177 (28.7)	164 (92.7)	13 (7.3)		
In couple	254 (41.2)	243 (95.7)	11 (4.3)		
Divorced/Widowed	19 (3.1)	13 (68.4)	6 (31.6)		
Group work experience				4.89	0.180
<5 years	171 (27.8)	163 (95.3	8 (4.7)		
5–10 years	178 (28.9)	192 (28.8)	192 (28.8)		
11–15 years	130 (21.1)	121 (93.1)	9 (6.9)		
>16 years	137 (22.2)	125 (91.2)	12 (34.3)		
Type of employment					
State / interim	44 (7.1)	40 (90.9)	4 (9.1)	8.55	0.036^*^
Freelancer	294 (47.7)	272 (92.5)	22 (7.5)		
Self-employed	262 (42.5)	255 (97.3)	7 (2.7)		
Not actives	16 (2.6)	14 (87.5)	2 (12.5)		
Professional role					
Employer	511 (83.0)	90 (92.8)	26 (5.1)	0.72	0.396
Employee/freelancer	97 (15.7)	485 (94.9)	7 (7.2)		
No answer	8 (1.3)				

*Data shown as number of responses (percentage)*.

* *p < 0.05 Chi-square calculated as contingency table*.

### Reasons Provided for Accepting the COVID-19 Vaccine

**Figure 2:**
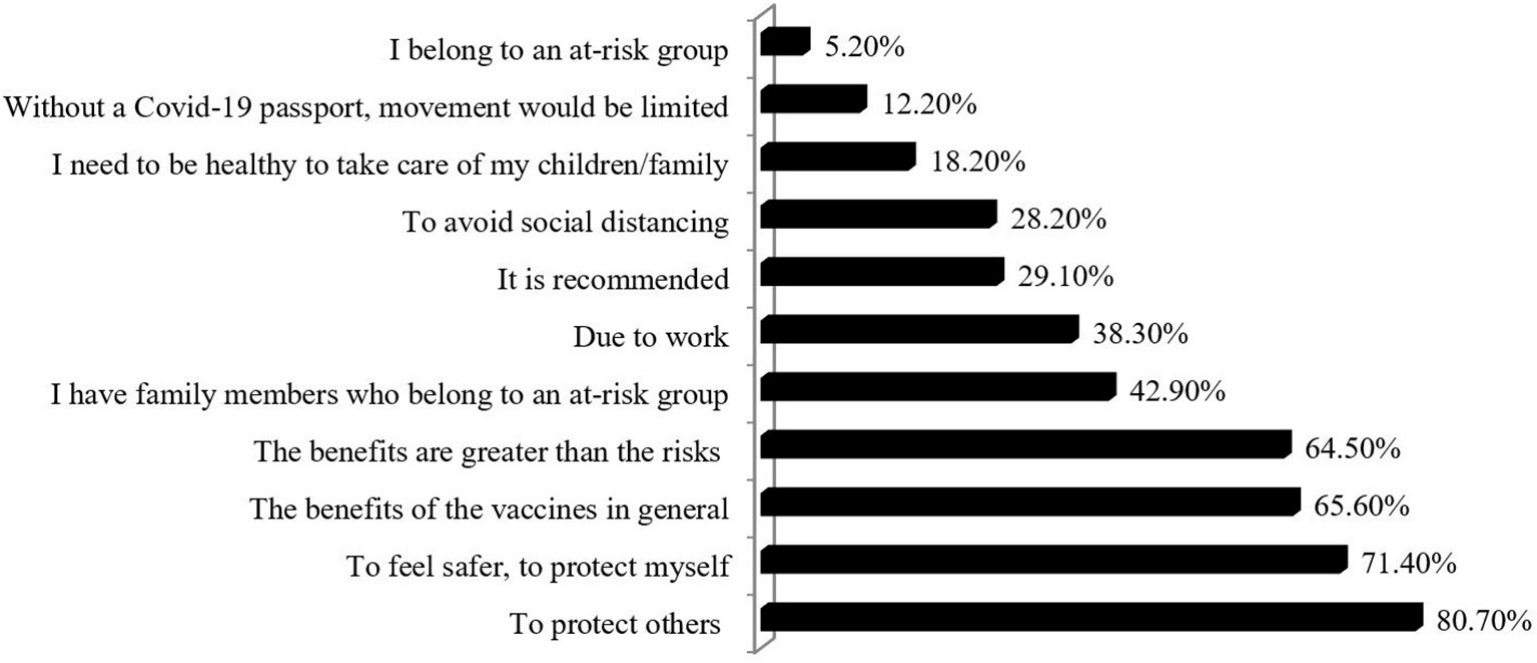
Reasons provided for COVID-19 vaccine acceptance.

The main reasons for vaccination were the need to protect others and oneself, along with the belief that vaccines were good for health. The fear of social distancing or the existence of a COVID-19 passport were not found among the main reasons for the vaccine acceptance of the physical therapists (Figure 2).

## Discussion

According to our knowledge, this is the first study conducted in Spain that evaluated the impact of the pandemic due to COVID-19 on physical therapists during the state of alarm decreed by the Spanish government, as well as the acceptance of the vaccine by this group of healthcare professionals.

### Impact on Work Among Spanish Physical Therapists

Among the participating physical therapists who were actively working, 62.1% indicated a reduction in the work schedule, with an economic impact of 5.9 ± 2.5 on a 10-point Likert scale. The reduction in the work schedule observed among the Spanish physical therapists is comparable to other countries such as the United States, where the clinical practices suffered a 60% decrease in the volume of patients and a 55% decrease in income due to COVID-19 (22, 23). Other examples include Iraq, where odontologists suffered economic losses of about 50% (24), and Romania, where the decrease in patients in the clinics consulted resulted in a decrease of their income of about 72.5% (21). This number increases to 100% in the Spanish odontology clinics (25).

The main reasons given for the decrease in patient care in consultations were to guarantee the safety of patients and worker (52.5%) (21, 23, 25). We must add the strict security measures imposed by the Government, and the scarcity or high prices of the protection equipment needed to meet these safety measures, which greatly aggravated the work and economic impact experienced by the clinics (26–29).

As shown in this study, most of the physical therapist professionals in Spain worked as freelancers or in small or medium enterprises (30). As the measures to contain the spread of the virus broadened, the economic impact on the private clinics was greater, and could be translated as loss of employment and qualitative and quantitative changes in care provision (31). In this sense, the physical therapists consulted who worked in the private sector indicated that their schedule was more reduced with respect to those who worked in the public sector. Also, during the state of alarm, physical therapists were considered as a basic service to treat only emergency situations, while the non-emergency healthcare activities that were scheduled were suspended (25, 32). This forced many private physical therapist clinics to close (36.4%), and most of the professionals (62.7%) had to ask for Government aid, of which 8% were rejected.

The emergency funds for freelancers, according to Decree 1/2020 and 44/2020 (33, 34), were destined to workers who were registered since December 31st, 2019 and with a reduction in income of 75%, with the individual quantity being between 750 and 1,500 in the case of many communities, such as the Community of Valencia, and which were compatible with the reductions in the Social Security contributions and the supplementary allowance due to cessation of activities (35).

Finally, self-employed workers who prove the total cessation or a 75% decrease in their activity compared to the previous quarter, may suspend or modify their supply contracts. Free medical insurance was offered from the different physiotherapist colleges that covered hospital admission for COVID-19 (36). However, the majority of those who received the aid (56.3%) stated that it was not enough to cover their needs, and only 12% indicated that the aid fully covered the economic impact during the state of alarm. For this reason, it is necessary to carry out an in-depth study to analyze the economic impact of the pandemic on private clinics and determine whether the emergency aid promoted by the Government was sufficient.

Another factor that influenced the reduction in the working hours of physical therapists in the private sector was the discomfort perceived by patients due to the security measures implemented in their clinics. As expected, the use of hydroalcoholic gel and masks, FFP2 and FFP3, were the most common measures (>90%), both in the public and private sectors. These measures were mandatory for both patients and health professionals, by mandate of the Ministry of Health (37). At the national level, these COVID prevention and control measures and the Vaccination Strategy applied to the socio-occupational situation of the individual have proven to be efficient and reach workers with the highest risk of COVID-19 early.

However, the physical therapists who worked in the private sector were more worried about their own health and that of their patients, and therefore implemented additional measures, such as the room disinfection devices using ozone or ultraviolet light. Working in private clinics was associated with the fear of having to close the clinic due to COVID-19 infection, which would perhaps lead to important economic losses, despite COVID-19 infection described in the Royal Decree 6/2020, from May 10th, as a common disease associated to a work accident, and the emergency measures adopted in the area of economy. This circumstance could have influenced the increased worry of the health professionals for avoiding infection of patients or employees in their private clinics, although this could result in an increase in the costs associated to the implementation of more safety measures in a period of economic recession.

### Acceptance of the Vaccine and the Attitudes of the Physical Therapists

The data showed a great acceptance of the first dose of the COVID-19 vaccine among the Spanish physical therapists consulted in June 2021 (87.2%). Like that observed in the general Spanish population (20), our study identified age, marital status, and employment as factors that influenced the decision of the physical therapists to accept or reject the COVID-19 vaccine. The high acceptance shown in our study was validated by a positive attitude among the physical therapists with respect to the belief of the protective effect of the COVID-19 vaccine and vaccines in general.

These results are similar as that obtained in May 2021 in Spain in a study conducted with 664 health professionals, whom, without differentiating between specialties, showed a vaccine acceptance rate of 79.4% (38). In both cases, the acceptance of the vaccine among Spanish health professionals was higher than that estimated in December 2020, in two studies conducted just before the start of the vaccination campaign (20, 39). In these previous studies, health workers were consulted on their intention to become vaccinated, with an estimation of acceptance of 52% among health professionals (20), specifically, an intention of vaccination of 82.5% among doctors, and 65.4% among nurses (39), always below the 87.2% found for the physical therapists who accepted the first dose in our study.

It seems to be the case that as the vaccination campaign progressed, the increase in the confidence of the COVID-19 vaccine among physical therapists increased, not only in Spain, but also in the rest of the world (40–42).

Among the adverse effects described by the physical therapists, the most frequent were pain in the area where the vaccine was given, tiredness, headache, as well as fever and generalized muscle pain. Just as our data show, localized pain after the injection seemed to be the most frequent adverse effect after the administration of the vaccine in the health professional population (40, 43, 44) as well as the general population (45–47).

The type of adverse effect observed among physical therapists was similar to that of other health professionals (41, 45, 47), although the frequency of appearance showed variations in some studies, especially if we compared them with the non-health professional population (48). We should take into account that it is less probable for health professionals to report the adverse effects at the beginning of the study, given their clinical experience and their ability to self-evaluate the severity of the disease (49). Only 9.1% of the vaccinated physical therapists indicated not suffering from any of the symptoms mentioned. This percentage is somewhat lower if we compare it to other health professionals such as Spanish nephrologists (25%) (40), although it is similar to health professionals from South Korea (7.3%) (44).

The two main brands administered to the participating Spanish physical therapists were AZ (61.1%) and PB (21.2%). Our study shows a greater appearance of adverse systemic effects such as headache (53.9%), shivering (51.2%) and generalized muscle pain (52.2%) in the physical therapists who received the AZ vaccine as compared to the other vaccines administered. This greater appearance of adverse effects was also found in other health workers who were vaccinated with the AZ vaccine as opposed to RNAm vaccines such as PB or Mo (39, 43).

Aside from the increased adverse effects, the administration of the AZ vaccine was associated with adverse events such as thrombocytopenia and thrombosis, which were not frequent although severe (49, 50). This situation led to the suspension of the vaccination campaign with the AZ vaccine in Spain due to the increase in the secondary effects described (17), and the decision of many countries to suspend its use. This damaged the perception of the safety of the AZ vaccine in Spain and surrounding countries (51). In our study, 3.5% of the physical therapists who received the first dose of AZ confirmed that they would not accept a second dose under any circumstance. This percentage of rejection was higher than that observed in Spain in May of 2021 among health professionals (2.1%) and the general population (1.5%) (38). This scenario of mistrust, mainly created by the adverse events associated to the AZ vaccine, led to the Spanish Ministry of Health agreeing that those younger than 60 who were vaccinated with one dose of AZ would be vaccinated, through a heterologous regime, with a second dose of PB, to avoid the appearance of thrombotic effects (52).

In contrast to the Spanish Government, the European Medicines Agency, supported by many Spanish health professionals through communication media, recommended the continuation with the AZ vaccine as a first and second dose at any age starting with 18 years of age, as the benefits were clearly greater than the adverse reactions (53). The results shown in the present study indicate that the physical therapists who received the first dose of the AZ vaccine would accept the second dose only in the case of having received the AZ vaccine (69.6%), and only 28 of the participants (24.3%) would accept another brand.

These data suggest that the physical therapists considered the scientific recommendations from the European Medicines Agency to a greater degree than those from the Spanish Government. This trust in favor of health and scientific institutions as opposed to the political positions, has been previously described among the Spanish population at the start of the vaccination campaign against COVID-19 (20). This demonstrates the importance of achieving a good coordination between the political and health institutions to provide information jointly, precisely, transparently, and correctly to the population, to guarantee a greater percentage of acceptance of the vaccine, which would allow reaching the immunity desired as soon as possible, to fight against the pandemic caused by COVID-19. This is valuable information to consider in future, similar scenarios.

### Limitations of the Study

Our study did not quantify the impact on work with respect to economic losses. The impact on work was evaluated from the consultation with health professionals regarding the decrease in scheduled appointments, the perceived economic impact and the factors that influence it. It would be necessary to carry out a quantitative study to obtain a numerical reference of the economic impact of the COVID-19 pandemic among physiotherapy clinics.

It is interesting that those working in the private sector compared to the public sector were more influenced by fear and discomfort of the new security measures, it could be a limitation that the study population was more than 90% physical therapists working in the private sector.

One of the limitations of our study was that a validated questionnaire was not found, which is not available in the scientific literature since COVID-19 is an emerging disease, in addition to the recall bias when reporting the symptoms that occurred in latest.

## Conclusions

The state of alarm decreed by the Spanish Government due to the COVID-19 pandemic had a negative impact on work and finances of public and private sector Spanish physical therapists. On the one hand, in the area of work, the modifications or the suspensions of the schedule appointments at private clinics, or the transfer of the health professionals to acute COVID-19 patient hospitals were some of the most notable (54).

On the other hand, in economic terms, the fear of infection of the patient and the imposition of safety measures compromised these private businesses. The aid offered by the Government was not enough to cover the needs of the physical therapists during the state of alarm period. Our study suggests the need to perform exhaustive qualitative analyses of the negative economic impact exerted by the pandemic, both in the past and presently, on the Spanish physical therapists during and after the pandemic. These studies could allow us to reflect on the efficacy of the aid offered to health professionals during the pandemic by the Government, to improve the actions taken during future, similar health emergencies, as well as to create new measures that will accelerate the post-pandemic economic recovery.

The vaccine was widely accepted by the Spanish physical therapists, improving the percentages estimated at the start of the vaccination campaign, and was not only influenced by the worry about their own health or that of the community at large, but by the economic impact that an infection in the work setting could signify.

However, the appearance of adverse effects during the vaccination campaign, and the discrepancy between political and health authorities when defining the vaccination strategy, increased the health professional's doubts for receiving the second dose. In this sense, the correct coordination between the different organizations and communication media during the vaccination campaign is indispensable for the health professionals to be able to offer the general population information that is precise, reliable, and trustworthy.

## Data Availability Statement

The raw data supporting the conclusions of this article will be made available by the authors, without undue reservation.

## Ethics Statement

The studies involving human participants were reviewed and approved by Ethics Committee from the CEU Cardenal-Herrera University (Refrence:CEI21/060). The patients/participants provided their written informed consent to participate in this study.

## Author Contributions

JS-M, JT, and NR-B: conceptualization and writing—review and editing. SM-N, JS-M, CS-A, CO-R, and JB-R: investigation and data curation. JS-M: statistical analysis. SM-N, JS-M, FM-P, and NR-B: writing—original draft. All authors contributed substantially to the study design data analysis and interpretation of the findings.

## Funding

This study recieved funding for open access publication fees from CEU-Cardenal Herrera University.

## Conflict of Interest

The authors declare that the research was conducted in the absence of any commercial or financial relationships that could be construed as a potential conflict of interest.

## Publisher's Note

All claims expressed in this article are solely those of the authors and do not necessarily represent those of their affiliated organizations, or those of the publisher, the editors and the reviewers. Any product that may be evaluated in this article, or claim that may be made by its manufacturer, is not guaranteed or endorsed by the publisher.

## Abbreviations

AZ: AstraZeneca-Oxford
CI: confidence interval
Mo: Moderna
OR: Odds ratio
PB: Pfizer-BioNTech
PPE: personal protection equipment
WHO: World Health Organization.
